# Evaluation of epithelial-to-mesenchymal transition and Ki-67 index in aggressive papillary thyroid cancer

**DOI:** 10.1016/j.bjorl.2024.101510

**Published:** 2024-09-10

**Authors:** Luana Perrone Camilo, Paula Vianna, Venancio Avancini Ferreira Alves, Beatriz Godoi Cavalheiro, Carlos Augusto Rossetti, Luiz Paulo Kowalski, Leandro Luongo Matos, Ana Kober Nogueira Leite

**Affiliations:** a Faculdade Israelita de Ciências da Saúde Albert Einstein, Hospital Albert Einstein, São Paulo, SP, Brazil; b Hospital Alemão Oswaldo Cruz, Laboratório de Patologia, São Paulo, SP, Brazil; c Faculdade de Medicina da Universidade de São Paulo, Instituto do Câncer do Estado de São Paulo, Laboratório de Investigação Médica 14 (LIM14), Departamento de Patologia, São Paulo, SP, Brazil; d Hospital das Clínicas da Faculdade de Medicina da Universidade de São Paulo, Instituto do Câncer do Estado de São Paulo, Departamento de Cirurgia de Cabeça e Pescoço, São Paulo, SP, Brazil

**Keywords:** Thyroid cancer, Papillary, Neoplasm metastasis, Immunohistochemistry

## Abstract

**Objectives:**

The present study aims to characterize immunohistochemical features of markers associated with Epithelial–Mesenchymal Transition (EMT) and proliferative activity that could lead to death in Papillary Thyroid Cancer (PTC).

**Methods:**

Clinical data and tumor material were retrospectively collected. The patients were separated into death from PTC (Group 1), metastatic cases with indolent behavior (Group 2) and non-metastatic indolent PTC (Group 3). Immunohistochemical assessment of E-cadherin, β-catenin, Vimentin, ZEB-1 and Ki-67 was performed in each tumor and a semiquantitative estimation of the percentage of expression was fulfilled on the best marking area at high of the tumor invasion front.

**Results:**

31 patients were included, 15 that died from PTC (Group 1), 6 in Group 2 and 10 in Group 3. The proliferative marker Ki-67 showed a significant difference in its expression in the tumor invasion front between the groups, specifically between groups 1 and 3 (*p* = 0.006). On the other hand, EMT-related immunohistochemical markers did not show significant difference in their percentage of expression, since loss of E-cadherin, β-catenin and Vimentin was observed in most cases at the invasion front.

**Conclusion:**

Patients that died from PTC had a significantly higher Ki-67 labelling index compared to patients with indolent disease (cutoff of 11%). Ki-67 may have a role as a prognostic marker and could be considered for routine use in PTC.

**Level of evidence:**

4.

## Highlights

• Despite being rare, death from the progression of papillary thyroid cancer occurs.
• Factors associated with this outcome are not completely understood.
• Ki-67 expression >11% was associated with death from the disease.

## Introduction

Thyroid cancer is the most prevalent endocrine malignancy with its prevalence increasing in the last three decades in the world.^1^ The most common type is Papillary Thyroid Carcinoma (PTC), a differentiated cancer that corresponds to approximately 85% of cases.^2^ PTC has an excellent prognosis, with the five-year survival rate reaching 99.8% for localized tumors and 97% for tumors with regional metastases.^3^ However, despite the low mortality, there are cases of deaths directly related to this cancer, with distant metastases as the main cause.

5%–25% of the patients with differentiated thyroid carcinomas present distant metastases and their five-year survival rate can be 57.3%.^3^ However, even amongst metastatic patients there is indolent behavior, with good disease control and even cases of complete response to radioiodine treatment.^4^

Although it is an extremely rare event, death from the progression of PTC occurs and the factors related to the difference in behavior in indolent and aggressive cases are not fully understood.^5^ The literature suggests that molecular changes are the main factors related to increasing aggressiveness and tumor invasion,^6^ however, no definitive prognostic markers were found in PTC. This differentiation is of extreme importance, especially now in the era of treatment de-escalation for PTC.^7^

There are different molecular pathways possibly related to cancer invasion and metastasis, one of them the Epithelial–Mesenchymal Transition (EMT).^8^ A complex network of environmental and transcription factor, as ZEB1, control the transition from a well-differentiated epithelial cell that express the adhesion molecule E-cadherin to a mesenchymal phenotype that is undifferentiated and express Vimentin.^9^ This process is associated with cancer invasion, metastasis, and aggressive behaviors. This pathway can be evaluated through immunohistochemical analyses, and therefore have a prognostic role in some cancers.^10^, ^11^, ^12^ However, the role of this process is scarcely explored in PTC literature.

The evaluation of EMT using IHC involves a combination of markers that highlight the loss of epithelial properties (e.g., E-cadherin, cytokeratins) and the gain of mesenchymal traits (e.g., Vimentin, N-cadherin, fibronectin). Additionally, transcription factors like Snail, Slug, ZEB1, ZEB2, and Twist, along with changes in β-catenin localization, provide insights into the regulatory mechanisms driving EMT. These markers collectively help in understanding the extent and nature of EMT in various biological and pathological contexts.^13^

Another marker of tumor aggressiveness is Ki-67. It is a nuclear antigen associated with the proliferative activity of intrinsic cell population in malignant tumors that can be immunohistochemically evaluated as a Labeling Index (LI). High expression of Ki-67 has consistently been associated with poor prognosis, advanced clinicopathological features, distant metastasis and an increased risk of disease recurrence in several cancer types,^14^, ^15^ but it is not used as a routine prognostic marker in PTC.^16^

Because of the indolent nature of the disease, PTC specimens usually have low Ki-67 LI compared to other tumors and therefore has been considered of limited use. However, higher Ki-67 expression in PTC has been associated with more aggressive behavior and poorer prognosis in some studies, but it is not consensus, and the association is mainly with disease recurrence and not with metastasis or mortality.^17^

Factors associated with tumor aggressiveness and especially cancer related deaths in PTC are a very difficult subject to study. The indolent nature of PTC and the rarity of deaths from the disease make it extremely difficult to obtain high quality data and large series on this specific outcome.

In this scenario, the present study aims to study the association of Ki-67 Labeling Index and EMT immunohistochemical markers with death from PTC.

## Methods

This is a retrospective case-control study including patients with papillary thyroid carcinoma who were treated with curative intention from January 2005 to January 2016 at Instituto do Câncer do Estado de São Paulo, Hospital das Clínicas, Faculdade de Medicina, Universidade de São Paulo (ICESP, HCFMUSP). The study was approved by the Institutional Review Board under the number 44997215.1.0000.0065.

All patients who died from the disease and had representative tumor material from formalin-fixed paraffin-embedded samples at the institution were included, and samples were selected as two control groups: (a) with patients that had non metastatic PTC followed for, at least, five years without recurrence and (b) patients that had distant metastasis with indolent course (without the disease progression for at least two years). The proportion of samples in each group was, respectively, 3:2:1. Therefore, the total number of patients included was 31, with 15 in the case group (death from PTC, Group 1) and 16 controls (6 with indolent distant metastasis — Group 2 and 10 with non-metastatic PTC – Group 3).

The tumors were staged according to the American Joint Committee on Cancer Tumor-Node-Metastasis (TNM) 8th edition.^18^ The data was collected and analyzed based on patient’s demographics, tumor characteristics, such as histologic type, size, extra-thyroid spread, recurrence, vascular invasion, nodal status, and extra-capsular spread. Data regarding treatment was also analyzed, with surgical information, use of radioiodine, external-beam radiation and/or chemotherapy, and the outcome, detailing the recurrence, distant metastases, cause of death and the period of each outcome.

Iodine avidity was determined by visual uptake at the known site of metastatic disease on WBS after the first Radioactive Iodine (RAI) adjuvant treatment under thyroid hormone stimulation. The tumor was considered refractory to RAI therapy based on the 2015 Guidelines of the American Thyroid Association^19^ as follows: malignant or metastatic tissue did not concentrate RAI, tumor tissue lost the ability to concentrate RAI following previous evidence of RAI-avid disease, RAI was concentrated in some lesions but not in others, and metastatic disease progressed despite significant RAI concentration.

The follow-up was done with clinical examination, laboratorial analyses of thyroid hormones, thyroglobulin, and anti-thyroglobulin antibody every six months, neck ultrasound, chest radiography and whole-body scan (selected cases) annually. The locoregional recurrence was confirmed by cytology, histology and imaging studies.

Immunohistochemistry was performed on 3 μm thick whole-tissue sections from each tumor with the following monoclonal antibodies: E-cadherin (clone 36B5, dilution 1:200, Leica Novocastra — Berlin, Berlin, Germany), β-catenin (clone MAB 14, dilution 1:400, BD — Franklin Lake, New Jersey, United States of America), Vimentin (clone V9, dilution 1:800, Genemed — South San Francisco, California, United States of America), ZEB-1 (clone CL0151, dilution 1: 1000, SIGMA — San Luis, Missouri, United States of America) and Ki-67 (monoclonal antibody MIB-1, dilution 1:50, Roche — Tucson, Arizona, United States of America). All reactions were subjected to signal amplification by the Novolink, Novocastra/Leika system and signal development with Diaminobenzidine and hydrogen peroxide.

In the absence of established protocols for quantifying the expression of proteins and transcription factors linked to EMT in PTC, a semi-quantitative estimation of the percentage of expression was performed using 10% increments and focusing on the best marking area (“hot spot”) at high magnification (400× Nikon eclipse E200 microscope) at the tumor invasion front. The percentage of nuclear positivity for KI-67 in 100 tumor cells at the tumor front was quantified on the best marking area (“hot spot”).

Specifically for E-cadherin and β-catenin, membrane staining was evaluated For Vimentin, the basal (subnuclear) cytoplasmic pattern was evaluated, and the presence of diffuse cytoplasmic labeling (mesenchymal phenotype) was also assessed. For ZEB-1 and Ki-67 the nuclear expression was evaluated.

Immunohistochemical variables were semiquantitated by two pathologists. No comparisons of performance were done, all questionable aspects were reviewed by both pathologists under a double-headed microscope, and the reported values were a result of a discussion and consensus.

Distributions were defined as parametric or non-parametric using the Kolmogorov–Smirnov test, according to the statistical program SPSS® version 29.0 (SPSS® Inc.; Illinois, USA). The values obtained by study of each continuous variable were described by means and Standard Error (SE) and/or by median and 95% Confidence Interval (95% CI).

Absolute and relative frequencies were used to describe qualitative data. To compare the means of two parametric sample populations, the Student “*t*” test was used. For three or more populations in non-parametric, the Kruskal–Wallis test with Dunn’s auxiliary test was applied. In validation as a diagnostic test, the ROC (Receiver Operating Characteristic) curve model was used.

## Results

There were fifteen thyroid tumor specimens from patients who died of disease progression in Group 1, six thyroid tumor specimens from patients with distant metastatic disease from indolent behavior (Group 2) and ten thyroid tumor specimens from patients with thyroid tumors restricted to the gland and non-metastatic in Group 3. Clinical, demographic, and pathological data for each group are described in Table 1.

**Table 1:** Clinical, demographic, and pathological data for each group.

Variable	Group 1 dead (n = 15)	Group 2 indolent metastasis (n = 6)	Group 3 non-metastatic (n = 10)
Gender			
Male	5 (33.3%)	3 (50%)	‒
Female	10 (66.7%)	3 (50%)	10 (100%)
Age at diagnosis (years old)	54 ± 12.8	55.5 ± 6.2	48.3 ± 3.8
Stimulated thyroglobulin (UI/mL)^a^	32,899.2 ± 72,760.8	3084.1 ± 2934.3	8.0 ± 4.5
Tumor size (cm)	5.0 ± 3.7	4.2 ± 1.1	3.7 ± 0.7
Radioiodine therapy	11 (73.3%)	6 (100%)	8 (80%)
Radioiodine refractory	8 (53.3%)	3 (50%)	0
Lymph node recurrence	7 (46.6%)	1 (16.7%)	0
Aggressive variant^b^	1 (6.6%)	0	0
TNM 8th edition			
T1	3 (20%)	1 (16.7%)	3 (33.3%)
T2	2 (13.3%)	1 (16.7%)	3 (33.3%)
T3	5 (33.3%)	2 (33.3%)	3 (33.3%)
T4A	2 (13.3%)	2 (33.3%)	‒
T4B	3 (20%)	‒	‒
N0	6 (40%)	2 (33.3%)	9 (90%)
N1A	3 (20%)	1 (16.7%)	1 (10%)
N1B	5 (33.3%)	3 (50%)	‒
Vascular invasion	11 (73.3%)	4 (66.7%)	4 (40%)
Microscopic extrathyroidal extension	2 (13.3%)	2 (33.3%)	3 (30%)
Macroscopic extrathyroidal extension	8 (53.3%)	3 (50%)	0
Multifocality	8 (53.3%)	4 (66.7%)	3 (30%)
Tumor dedifferentiation	8 (53.3%)	0	0

a Stimulated thyroglobulin is measured at the time of radioiodine therapy (postoperative).

b Columnar cell, tall cell, diffuse sclerosing or hobnail variants.

In Group 1, all patients had distant metastases, with 10 cases (66.7%) of synchronous diagnosis with the primary tumor. The most frequently affected site was the lung (14 cases, 93.7%), followed by bone (11 cases, 73.2%) and liver (4 cases, 26.6%). Of them, thirteen patients (86.7%) had metastases in more than one site and 12 (80%) received some specific treatment in addition to iodine for metastases (targeted therapy, surgery, or radiotherapy). Among the six metastatic cases in Group 2, three diagnoses were synchronous with the primary tumor (50%) and all cases had lung metastases. Only one case (16.7%) had metastasis in more than one site, affecting the lung and bone.

In Group 1, the mean time between diagnosis and death was 56.2 ± 9.8 months (range, 4–155 months). In Group 2, the mean follow-up time was 148.2 ± 16.5 months (119–204 months) and in Group 3, 117.2 ± 8.6 months (72–151 months).

In Table 2, the means of percentage of the immunohistochemical expression markers at the invasion front are described.

**Table 2:** Expression of immunohistochemical markers.

Marker	Group 1 dead (n = 15)		Group 2 indolent metastatic (n = 6)		Group 3 non-metastatic (n = 10)		*p-*Value (Kruskal–Wallis)
	Mean	SE	Mean	SE	Mean	SE	
Vimentin %	42.1	8.7	50	14.8	50	12.7	0.77
Ki-67 LI%	13.8	2.1	9	2.2	3.8	1.2	0.006
E-cadherin	46.4	5.8	40	10.3	51.5	9.8	0.675
β-catenin	45.7	8.5	0	16.6	66	9.7	0.297
ZEB-1	50.3	6.8	41.6	11.0	47	8.3	0.748

LI, Labeling Index.

EMT-related immunohistochemical markers showed no significant difference in their expression between the different groups. Loss of E-cadherin, β-catenin and Vimentin was observed in most cases of all groups at the invasion front, as well as the increased diffuse cytoplasmic expression (mesenchymal phenotype) of Vimentin. On the other hand, the Ki-67 marker showed a significant difference (*p* = 0.006) between case and control groups.

**Figure 1:**
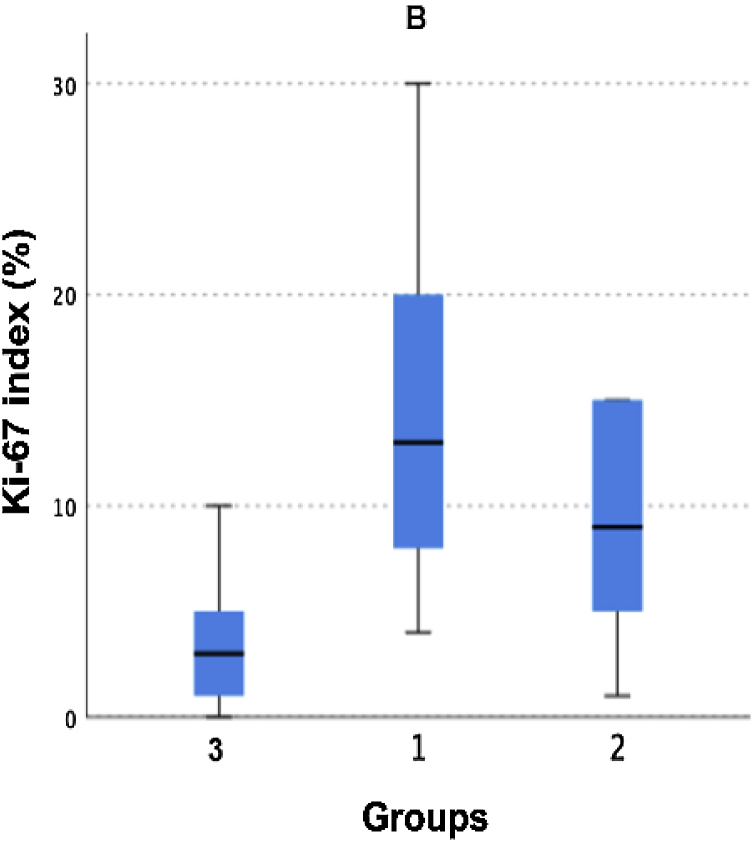
Box-plot graph illustrating the difference in Ki-67 marker expression between groups. Group 1: dead; Group 2: indolent metastatic; Group 3: non-metastatic.

For the Ki-67 marker, which showed a significant difference in its expression between the three groups in the Kruskal–Wallis analysis, a pairwise analysis demonstrated a significant difference in Ki-67 expression between groups 1 (dead) and 3 (non-metastatic), with *p* = 0.001 (Table 3). The graphic expression of the difference in expression between the groups is shown in Fig. 1.

**Table 3:** Ki-67 marker expression, pairwise comparison.

Group	*p* (Dunn’s auxiliary test)
Groups: 1 (dead) vs. 2 (indolent metastatic)	0.332
Groups: 3 (non-metastatic) vs. 2 (indolent metastatic)	0.09
Groups: 1 (dead) vs. 3 (non-metastatic)	0.001

**Figure 2:**
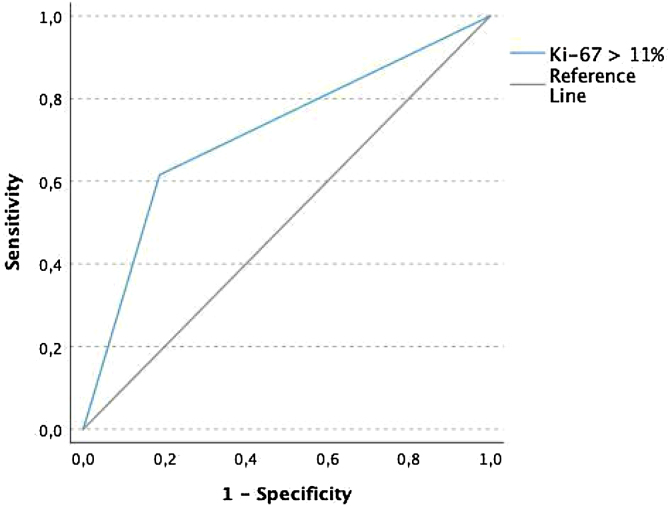
ROC curve demonstrating the accuracy of Ki-67 LI > 10% to diagnose death due to PTC progression (sensitivity 0.61, specificity 0.87, and area under the ROC curve 0.71).

To determine the cutoff value for the Ki-67 LI a ROC curve analyses was performed and the determined LI cutoff was established in 11% for the diagnostic of death due to PTC progression (sensitivity 0.61, specificity 0.87, and area under the ROC curve 0.71; Fig. 2).

## Discussion

The present study is the first to our knowledge to study the role of EMT and Ki-67 immunohistochemical markers in PTC-related death in the Brazilian population.

The EMT process was observed in all groups at the invasive front, characterized by the lost membranous immunohistochemical expression of E-cadherin and β-catenin, while, in contrast, displayed an increased diffuse cytoplasmic expression (mesenchymal phenotype) of Vimentin.

There was no significant difference in the expression of EMT-related markers between groups with different clinical behaviors, which may be attributed to several factors. Although the role of EMT in oncogenesis is widely accepted, the process remains extremely complex and not fully understood, as it is not always associated with invasive behavior and is scarcely explored in Papillary Thyroid Carcinoma (PTC). Additionally, various methods exist to evaluate EMT in tumors, with immunohistochemical analysis being one option.

Vimentin, E-cadherin, and β-catenin are the most frequently described markers in the literature, but other markers such as N-cadherin, Snail, Twist1, TGF-β, and VEGF are also utilized. The selection of these specific markers was based on their high sensitivity for identifying EMT, using well-established and reliable antibodies. Each marker evaluates a distinct aspect of the EMT process, as recommended in the guidelines.^13^

The lack of association between EMT markers and clinical behavior may indicate that EMT is not the main pathway related to aggressive behavior in the rare cases of death from PTC. Alternatively, it may be related to the limitations of the current series and methodology. Since a lack of association in a limited sample size should not be interpreted as evidence that this association does not exist, further studies are needed to explore the role of the complex EMT process in aggressive papillary thyroid cancer.

In the present study, patients that died from the disease had a significantly higher Ki-67 LI compared to indolent tumors, with 11% established as the cutoff. There are studies^20^, ^21^, ^22^ in literature exploring the role of Ki-67 in PTC but the majority are regarding its association with recurrence and lymph node metastasis.

Lei et al.^20^ analyzed 430 PTC specimens and identified a positive association of Ki-67 LI > 5% with lymph node metastasis and worse Disease-Free Survival (DFS). Matsuse et al.^21^ studied the association of TERT promoter mutation and Ki-67 LI with recurrence and identified that a LI > 10% was associated with an increased risk of recurrence by 5.5 times. Lindfors et al.^22^ on the other hand, established an association of a LI > 3% with lower disease free survival.

Ito et al.^17^ investigated the prognostic value of Ki-67 in 371 patients with PTC. Ki-67 LI was associated with patient age, massive extrathyroid extension, and distant metastasis at surgery. Also, Ki-67 LI > 1% (HR = 4.13, 95% IC 2.19–7.75) was identified as an independent prognostic factor for lower DFS, together with advanced age, N1b, and massive extrathyroid extension. The authors found that all patients that died had a Ki-67 LI > 1% and on multivariate analyses Ki-67 LI > 3% was associated with cause specific survival (HR = 25.64 95% IC 2.49–250). Moreover, all patients that died had a KI-67 LI ≥ 4%. However, in this series, only eight patients died from the disease, and this low mortality represents the main difficulty in studying factors related to death in PTC.

Another study^23^ found a significant association between Ki-67 LI and disease-specific mortality in patients with PTC. The rates of disease-specific survival at 10 years were reported to be significantly different among patients with Ki-67 LI ≤ 5%, 5%–10%, and >10%, with higher Ki-67 LI values correlating with lower survival rates. Additionally, Ki-67 LI > 10% was identified as an independent factor for disease-specific survival in multivariate analyses (HR = 34, 95% CI 3.8‒305, *p* < 0.01). This series, however, only had 6 patients that died from PTC.

The study of mortality and risk factors for death in papillary carcinoma, an indolent neoplasm with very low mortality, is extremely complex due to the scarcity of patients and the need of very long follow up periods.

In an effort to address this difficulty and validate our findings in a larger cohort, we analyzed data from The Cancer Genome Atlas (TCGA)^24^ using the R2: Genomics Analysis and Visualization Platform (http://r2.amc.nl) and The Human Protein Atlas platform (https://www.proteinatlas.org/). This database includes samples from 501 PTC cases with available protein expression data. Among these patients, the expression levels of Vimentin, E-cadherin, β-catenin, ZEB-1, and Ki-67 did not show significant differences between those who were alive and those who had deceased. However, it is important to note that this database contains only 16 deceased patients, with only 6 confirmed to have died with the disease. Thus, even with a large database, the number of patients who died from PTC remains limited, highlighting the rarity and significance of our cases. Additionally, the highest Ki-67 Labeling Index (LI) reported in this series was 5.1%, suggesting that this cohort does not include PTC cases with truly aggressive behavior. Therefore, even though the present study has a small sample, it is of great importance because it confirms Ki-67 as a factor associated with PTC related death.

Including patients with distant metastasis but with indolent behavior in the analyses provides some interesting insights. There was no significant difference in Ki-67 expression between this group and any of the others. This suggests that higher Ki-67 expression is indeed related to mortality and truly aggressive behavior, rather than to indolent disease, even when it is widespread.

The use of Ki-67 marker in thyroid cancer specimens is not recommended in current guidelines, nor is it used routinely in clinical practice.^19^, ^25^ However, as discussed, there is evidence that it could have a role as a prognostic marker and maybe should be considered for routine use in PTC as it is in other cancer types.^26^ In the era of de-escalation of treatment for PTC, the identification of prognostic factors is also of great importance so more aggressive treatment can be reserved for more aggressive tumors.

This study is limited by its retrospective nature and small sample size, which constitutes its most significant limitation. However, given the rarity of death from PTC, this series represents the largest analysis of Ki-67 Labeling Index (LI) in tumors from patients who died from PTC, underscoring its importance. Future studies could include additional EMT markers to further explore this process. Although the expression of EMT-related immunohistochemical markers did not show a significant difference between the groups in this study, the observed difference in the expression of the Ki-67 marker is of great clinical value and has been scarcely explored in PTC.

## Conclusion

Patients that died from PTC had a significantly higher Ki-67 LI compared to patients with indolent disease.

## Authors’ contributions

Luana Perrone Camilo, Ana Kober Nogueira Leite: Conceptualization, Data curation, Formal analysis, Investigation, Methodology, Project administration, Writing original – draft and Writing – review and editing. Leandro Luongo de Matos: Conceptualization, Data curation, Statistical analysis, Writing – review and editing. Paula Vianna, Venancio Avancini Ferreira Alves: Data curation, Immunohistochemical analysis, Writing – review and editing. Luiz Paulo Kowalski, Beatriz Godoi Cavalheiro, Carlos Augusto Rossetti: Investigation, Data collection, Writing – review and editing. All authors participated in meetings every 15 days about the progress of the research and the manuscript.

## Funding

The present study was supported by the Fundação de Amparo à Pesquisa do Estado de São Paulo (10.13039/501100001807FAPESP) ‒ Finance Code 2020/12459-1 ‒ Grant to L.P.C.

## Conflicts of interest

The authors declare no conflicts of interest.
